# Short periods of bipolar anodal TDCS induce no instantaneous dose-dependent increase in cerebral blood flow in the targeted human motor cortex

**DOI:** 10.1038/s41598-022-13091-7

**Published:** 2022-06-10

**Authors:** Marie Louise Liu, Anke Ninija Karabanov, Marjolein Piek, Esben Thade Petersen, Axel Thielscher, Hartwig Roman Siebner

**Affiliations:** 1 Danish Research Centre for Magnetic Resonance, Centre for Functional and Diagnostic Imaging and Research, Copenhagen University Hospital Amager and Hvidovre, Hvidovre, Denmark; 2 Department of Nutrition, Exercise and Sports, University of Copenhagen, Copenhagen, Denmark; 3 Department of Health Technology, Technical University of Denmark, Kongens Lyngby, Denmark; 4 Department of Neurology, Copenhagen University Hospital Bispebjerg, Copenhagen, Denmark; 5 Department of Clinical Medicine, Faculty of Medical and Health Sciences, University of Copenhagen, Copenhagen, Denmark

**Keywords:** Neuroscience, Motor control, Motor cortex

## Abstract

Anodal transcranial direct current stimulation (aTDCS) of primary motor hand area (M1-HAND) can enhance corticomotor excitability, but it is still unknown which current intensity produces the strongest effect on intrinsic neural firing rates and synaptic activity. Magnetic resonance imaging (MRI) combined with pseudo-continuous Arterial Spin Labeling (pcASL MRI) can map regional cortical blood flow (rCBF). The measured rCBF signal is sensitive to regional changes in neuronal activity due to neurovascular coupling. Therefore, concurrent TDCS and pcASL MRI may reveal the relationship between current intensity and TDCS-induced changes in overall firing rates and synaptic activity in the cortical target. Here we employed pcASL MRI to map acute rCBF changes during short-duration aTDCS of left M1-HAND. Using the rCBF response as a proxy for regional neuronal activity, we investigated if short-duration aTDCS produces an instantaneous dose-dependent rCBF increase in the targeted M1-HAND that may be useful for individual dosing. Nine healthy right-handed participants received 30 s of aTDCS at 0.5, 1.0, 1.5, and 2.0 mA with the anode placed over left M1-HAND and cathode over the right supraorbital region. Concurrent pcASL MRI at 3 T probed TDCS-related rCBF changes in the targeted M1-HAND. Movement-induced rCBF changes were also assessed. Apart from a subtle increase in rCBF at 0.5 mA, short-duration aTDCS did not modulate rCBF in the M1-HAND relative to no-stimulation periods. None of the participants showed a dose-dependent increase in rCBF during aTDCS, even after accounting for individual differences in TDCS-induced electrical field strength. In contrast, finger movements led to robust activation of left M1-HAND before and after aTDCS. Short-duration bipolar aTDCS does not produce consistant instantaneous dose-dependent rCBF increases in the targeted M1-HAND at conventional intensity ranges. Therefore, the regional hemodynamic response profile to short-duration aTDCS may not be suited to inform individual dosing of TDCS intensity.

## Introduction

Transcranial Direct Current Stimulation (TDCS) is widely used to modulate cortical function in cognitive neuroscience and in therapeutic settings^1–8^. The underlying mechanism of action still remains to be fully clarified. Neurophysiological studies suggest that TDCS modulates the intrinsic activity of cortical neurons by inducing shifts in the neuronal membrane potential, presumably by polarizing axon terminals^9,10^.

TDCS has been shown to produce polarity-specific bi-directional effects on corticomotor excitability by using a bipolar montage with one skin electrode placed on the scalp over the primary motor hand area (M1-HAND) and the other electrode placed on the contralateral supraorbital region^11–13^. However, high inter-individual variability has been observed in neurophysiological studies using single-pulse Transcranial Magnetic Stimulation (TMS) and motor evoked potential (MEP) to probe lasting effects of TDCS on corticospinal excitability^14^, and the consistency of the after-effects has been challenged by several recent studies^15–17^. This is also reflected in therapeutic settings, were the majority of TDCS studies have yielded varying results and have failed to provide clear evidence for efficacy^18^.

The large inter-individual variability may partially be caused by the way current intensity of TDCS is determined. Differences in head and brain anatomy significantly influence strength and spatial distribution of the induced electrical field (E-field)^19,20^. Adjusting the stimulation intensity using field simulations informed by individual structural MRI can account for some variance at the single-person level^21,22^, but we still lack personalized “dosing methods” that allow to adjust the current intensity of TDCS based on the individual cortical response profile. Personalized dosing requires reliable brain mapping approaches that can reliably delineate the dose–response relationship between the TDCS current intensity and the TDCS induced change in regional cortical activity at the single-person level. This information might help to personalize the current intensity of TDCS in a way that the induced E-fields optimally engages the targeted cortical area.

For dose–response mapping, we were interested in the acute and immediate changes in neural activity induced by short periods of TDCS, as they reflect the direct impact of stimulation in the cortical target region, in contrast to the aftereffects of prolonged stimulation that can be influenced by secondary mechanisms over several minutes.

### Short period TDCS inducing immediate neuronal effects

Previous work has used single-pulse TMS to demonstrate that short periods of bipolar TDCS of M1-HAND evokes acute changes in corticospinal excitability. Early TDCS studies used TMS to show that short periods of bipolar anodal TDCS (aTDCS) (4–120 s) targeting M1-HAND induce an immediate rise in MEP amplitude, indicating an instantaneous rise in corticospinal excitability without sustained after-effects^11,23^. Single-pulse TMS also revealed an acute increase in the amplitude of the motor evoked responses in the contralateral hand during 30 s of aTDCS at a current intensity of 1.5 mA^24^. In that study, the acute “online” effect was also predictive of the lasting increase in MEP amplitude after the end of aTDCS^24^. Given the neurophysiological evidence that short periods of aTDCS do cause a shift in corticospinal excitability, we decided to use short stimulation periods of 30 s during pcASL MRI in order to map the relationship between the current intensity of TDCS and the magnitude of the regional rCBF response in the targeted M1-HAND.

### Acute perfusion response to brain stimulation

Short periods of transcranial magnetic stimulation (TMS) have been shown to induce an acute modulation of regional blood flow in the targeted motor cortex. For instance, Siebner et al. used H_2_^15^O positron emission tomography (PET) in six healthy individuals to show a consistent dose–response pattern during subthreshold repetitive TMS of the left M1-HAND^25^. All participants showed a linear increase in regional cerebral blood flow (rCBF), when the frequency of repetitive TMS was gradually increased from 1 to 5 Hz^25^. Another study by Paus et al. stimulated the frontal eye field of six healthy individuals with TMS during H_2_^15^O PET and found a relative increase in regional blood flow in the stimulated region with the number of stimuli that were applied during the PET scan. Robust immediate and short-term rCBF changes in the stimulated sensorimotor cortex had also been found in another H_2_^15^O-PET study, targeting the left M1-HAND with sub-motor threshold TMS at 5 Hz^26^.

Several preclinical studies have also verified that the acute exposure to short periods of direct-current stimulation gives rise to instantaneous changes in spontaneous neural firing and regional blood flow in the stimulated cortex. Direct electrophysiological intracortical recordings showed that short periods of radially applied anodal direct current stimulation raises the mean firing rate of cortical neurons, with opposite effects during cathodal direct current stimulation^27–29^. While we are not aware of any preclinical study that has measured the acute vascular response to short periods of TDCS, prolonged periods of TDCS induced polarity-specific after-effects on cortical blood perfusion in the rat^30^. Yet there is evidence for immediate changes in rCBF during short periods of pulsed or alternating current stimulation^31–34^.

### TDCS and perfusion imaging studies

One promising way to map the regional neurovascular response to TDCS is with functional magnetic resonance imaging (fMRI) and Arterial Spin Labelling (ASL), that uses magnetically labeled water in the arterial blood as a tracer of rCBF^35,36^. Despite of the evidence for immediate neurovascular effects of electric brain stimulation, fMRI studies focusing on immediate TDCS effects are sparse^37–39^. A few perfusion studies using ASL-fMRI and H_2_^15^O -PET measuring sustained after-effects of TDCS with longer stimulation periods indicate a general increase of rCBF during bipolar TDCS^40–42^ and recent ASL-fMRI studies show dose-related increases in rCBF in the cortex underneath the precentral electrode during and after aTDCS at conventional intensities (0.5–2.0 mA)^43^ and at higher intensities up to 4 mA^44^.

Prompted by the previous combined TMS-PET studies and TDCS ASL-fMRI studies, we measured regional brain perfusion with MRI to test whether short duration TDCS would also induce consistent dose–response patterns on a single subject level. Using a classical bipolar electrode arrangement, we applied 30 s epochs of aTDCS at four different intensities from 0.5 to 2.0 mA to the left M1-HAND^45^. Simultaneously, we measured regional cortical perfusion with pseudo-continuous arterial spin labelling (pcASL). We expected that all individuals would show, at least to some degree, an instantaneous and intensity dependent pattern of regional perfusion changes in the target cortex. If this were the case, we reasoned that ASL-fMRI might be used to adjust the individual intensity of TDCS in future studies.

## Results

None of the participants experienced major side effects during the study. The results of the psychometric assessment are presented in Appendix A as Supplementary Material.

### Regional perfusion changes in the precentral M1-HAND target region

**Figure 1:**
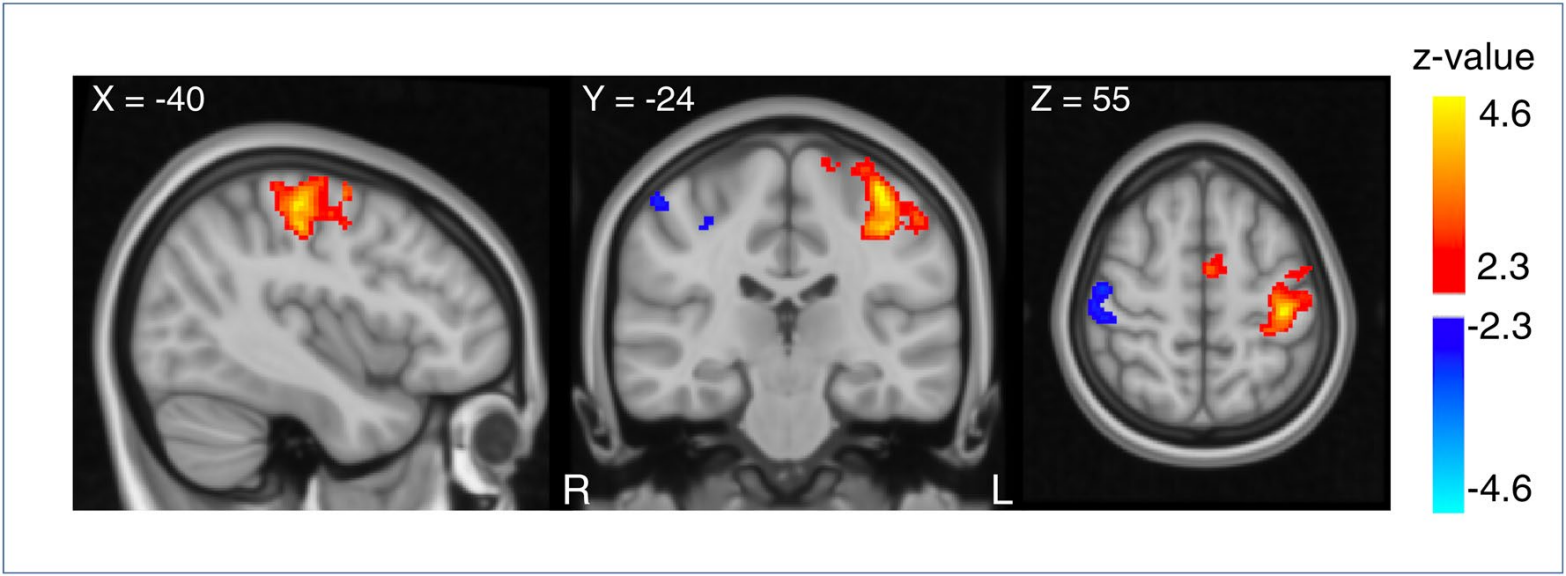
Perfusion changes during right hand finger-tapping before stimulation (FT-pre) on all subjects. Regions show changes in perfusion during FT compared to resting state. Subjects show increase in perfusion corresponding to the left sensorimotor cortex (red), (Cluster size = 1294, Z-max = 4.68, peak coordinate: − 38, − 24, 60, cluster p-value = 1.72e−08) and decreased perfusion corresponding to the right sensorimotor cortex (blue) (Cluster size = 244, Z-max = 3.0, peak coordinate: 50, − 14, 54, cluster p-value = 0.047). Results are overlaid on MNI template. The figure shows the results for a voxel-wise threshold at p = 0.01 (Z = 2.3) and cluster corrected threshold of p = 0.05 for illustrative purposes. Perfusion changes during tDCS did not survive the cluster-corrected level. The primary analysis was performed with a cluster-defining threshold of p = 0.001 (Z = 3.1) at the voxel level and cluster corrected threshold of p = 0.05 (shown in Supplementary Fig. 2).

**Figure 2:**
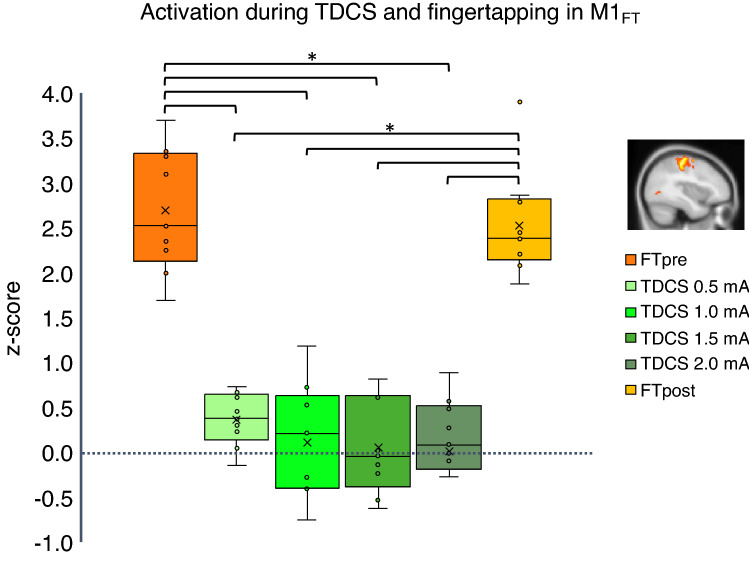
Change in regional cerebral blood flow in left primary motor cortex. The region is defined corresponding to the regional activation during the finger-tapping task prior to stimulation (FTpre) as shown red colors in Fig. 1. Box and whisker plot of activation during TDCS at 0.5–2.0 mA (green shades) and finger-tapping before stimulation (FTpre) and after (FTpost) (orange shades). Y-axis shows activation in z-score. Outliers defined being beyond 1.5 inter-quartile range of each quartile.

We first examined mean perfusion changes during finger-tapping prior (FTpre) and post stimulation (FTpost) and during aTDCS from 0.5 to 2.0 mA within the volume of interest (VOI) defined by the finger-tapping related functional activation, M1_FT_ (Illustrated in Fig. 1, and Supplementary Fig. 2. (For more details on our VOI definitions, we refer to the methods section and Table 3). Two-way repeated measures ANOVA revealed a significant main effect of “conditions”, indicating a difference in mean rCBF within M1_FT_ between all six experimental conditions (FTpre, FTpost, 0.5 mA TDCS, 1.0 mA TDCS, 1.5 mA TDCS, 2.0 mA TDCS) (F = 54.205, p < 0.000). Pairwise comparisons showed a significantly higher rCBF during FT compared to all aTDCS intensities after Bonferroni correction (Table 1). FT induced rCBF increases after the aTDCS blocks did not differ from FT induced perfusion prior to aTDCS (FTpost vs. FTpre). There was no significant difference in rCBF between any of the applied aTDCS intensities, but mean rCBF during 0.5 mA aTDCS was slightly higher than baseline rCBF (Fig. 2). Post-hoc exploratory one-sample t-tests on each intensity condition revealed an increased perfusion (z-score) in M1_FT_ at 0.5 mA aTDCS compared to baseline, p_uncorrected_ = 0.005 which survived Bonferroni correction (p_Bonferroni_ = 0.02).

**Table 1:**
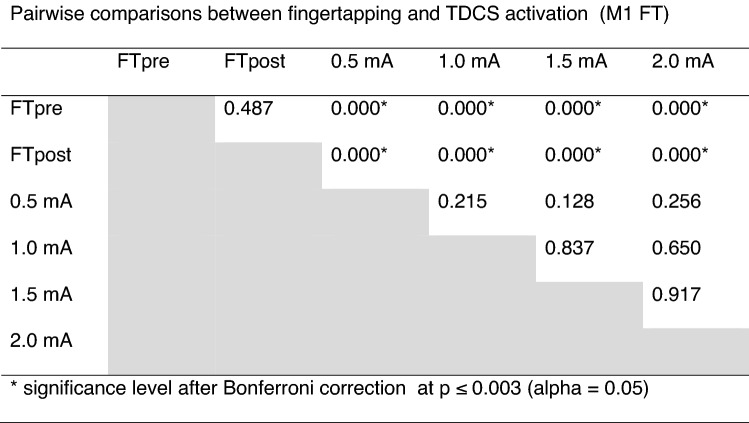
Pairwise comparisons between fingertapping and TDCS activation (M1 FT).

**Figure 3:**
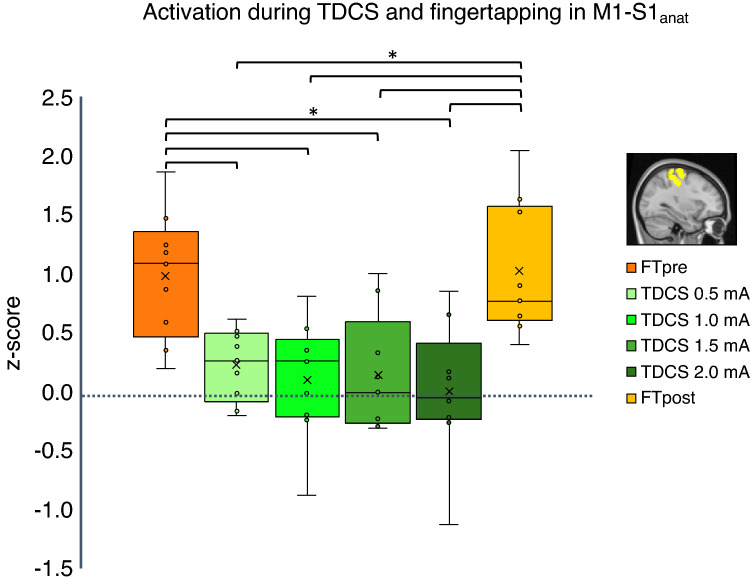
Change in regional cerebral blood flow in left primary sensorimotor cortex. The region is defined using the probabilistic anatomical Juelich atlas. Areas BA4a, BA4b BA3a, BA3b and BA6 for the left hemisphere was used, and the mask was limited in each direction to fit the hand knob. Box and whisker plot of activation during TDCS at 0.5–2.0 mA (green shades) and finger-tapping before stimulation (FTpre) and after (FTpost) (orange shades). Y-axis shows activation in z-score. Outliers defined being beyond 1.5 inter-quartile range of each quartile.

To control for any potential bias introduced by using a group-specific functional VOI (M1_FT_), we also analyzed rCBF changes using an anatomically defined VOI (M1-S1_anat_) (Fig. 3).

The rCBF changes in the M1-S1_anat_VOI were less strong compared to M1_FT_, but revealed a similar response pattern. Two-way repeated measures ANOVA revealed a significant main effect of “conditions” (F = 9.436, p < 0.000). Pairwise comparisons showed significant difference between rCBF during FT compared to all stimulation intensities after Bonferroni correction (Table 2), with no difference between FTpost vs. FTpre and no significant difference in rCBF between the aTDCS intensities. One-sample t-test on each aTDCS intensity also revealed increased perfusion at 0.5 mA compared to baseline, p_uncorrected_ = 0.028, but the increase in rCBF during aTDCS at 0.5 mA did not survive correction for multiple comparisons (p_Bonferroni_ = 0.112).

**Table 2:**
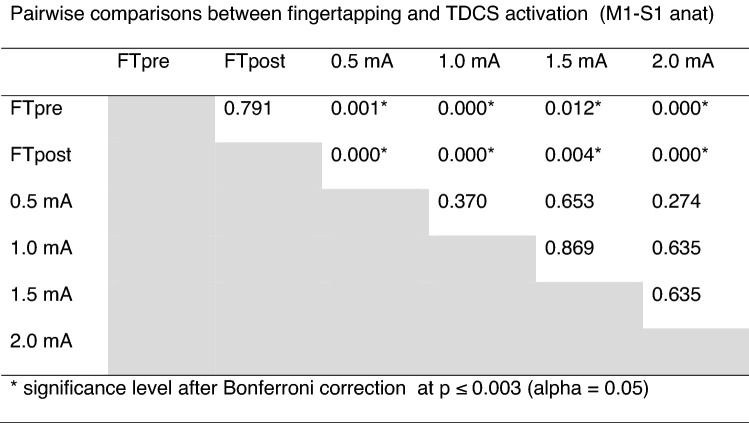
Pairwise comparisons between fingertapping and TDCS activation (M1-S1 anat).

**Figure 4:**
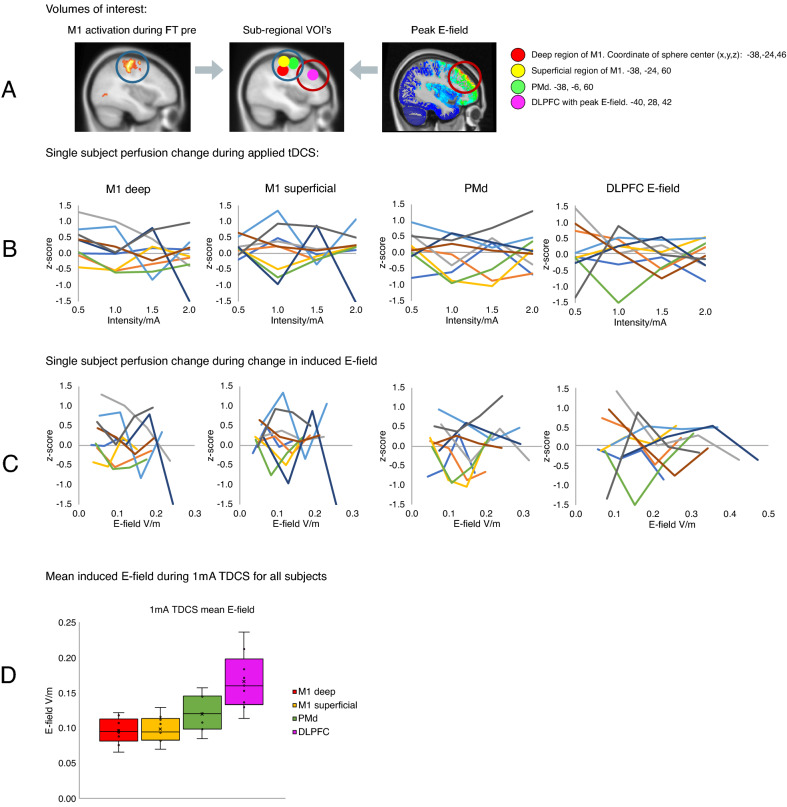
Individual changes in regional cerebral blood flow within four spherical volumes of interest (VOIs) with a radius of 10 mm. (**A**) The regional peak activations during finger-tapping prior to stimulation (FT-pre) was used as a functional localizer for the three spheres. Spheres were placed in the superficial primary motor cortex (M1) (yellow, center coordinate: − 38; − 24; 60) the deep part of M1 (red, center coordinate: − 38; − 24; 46) and the left dorsal premotor cortex (green, center coordinates − 38; − 6; 60). The fourth VOI (magenta) was placed at the location of maximum induced E-field in all subjects (center coordinate − 40; 28; 42). (**B**) Plots show individual activation within each VOI for each stimulation intensity. (**C**) Plots show individual activation within each VOI as a function of the induced E-field instead of TDCS intensity. (**D**) Boxplot of mean induced E-field in all four VOI’s at 1 mA TDCS. Y-axis shows induced E-field V/m. Outliers defined being beyond 1.5 inter-quartile range of each quartile.

Analysis of aTDCS-related perfusion changes in the sub-regions M1_deep_, M1_superficial_ and PMd (VOI’s shown in Fig. 4A) revealed no significant main effect of aTDCS intensity on rCBF in any of the functionally defined sub-regional VOIs (VOI_M1deep_: F = 1.04 p = 0.38, VOI_M1superficial_: F = 0.33 p = 0.81, VOI_PMd_: F = 0.63 p = 0.61).

### Perfusion changes in the cortical region exposed to the highest E-field

The aTDCS-induced electrical field reached its maximum in the dorsolateral prefrontal cortex (DLPFC_E-field_) (Fig. 4A). The mean E-field in the dorsolateral prefrontal VOI (DLPFC_E-field_) was consistently higher than the mean E-field in the precentral VOIs (Fig. 4D). We did not find any significant effect of aTDCS or FT on mean rCBF in the DLPFC_E-field_ VOI (F = 0.290 p = 0.916) and individual rCBF activity in the E-field guided prefrontal VOI was highly variable, showing no consistent intensity dependent response pattern (Fig. 4B).

### Whole-brain analysis

Whole-brain analysis revealed no significant increase in rCBF for any of the four aTDCS conditions, or any linear voxel-wise increase or decrease. This was different for the finger tapping task. Right-hand FT before aTDCS (FTpre) lead to increase in rCBF in left M1-HAND (Fig. 1 and Supplementary Fig. 2). Exploratory analysis at a more liberal cluster-forming threshold (z = 2.3 or p = 0.01) also showed a tapping-associated decrease of rCBF in the right primary sensorimotor cortex ipsilateral to the tapping right hand (Fig. 1). FT after stimulation (FTpost) with the right hand also led to increase in rCBF in left M1-HAND (Supplementary Fig. 2).

### Relationship between induced E-field and regional perfusion changes

Neither for M1_superficial_, M1_deep_, PMd and or DLPFC_E-field_, we found a consistent relationship between regional perfusion changes and the induced E-field in our participants (Fig. 4C). We tested whether the perfusion levels in these VOIs could be predicted by individual induced E-field by specifying a linear regression model to predict perfusion activity (z-score) from E-field at each of the four aTDCS intensities. Only the E-fields induced with aTDCS at an intensity of 0.5 mA significantly predicted z-score in M1_deep_, F = 9.333, p_Uncorrected_ = 0.018, R^2^ = 0.571. This however did not survive Bonferroni correction for multiple comparisons (p_Bonferroni_ = 0.288). The corresponding plots are shown in Supplementary Fig. 4.

## Discussion

Using pcASL-MRI, we measured relative changes in rCBF as proxy for regional neural activity during short epochs of bipolar aTDCS targeting the left M1-HAND. In different aTDCS blocks, we varied the intensity of aTDCS, applying currents at 0.5, 1.0. 1.5, and 2.0 mA. We found no evidence in support of the hypothesis that aTDCS produces intensity-dependent increases in rCBF in the cortical target region. This was the case for all subregions of the targeted pericentral cortex but also in the portion of the laterodorsal prefrontal cortex where the induced E-field was highest.

### No modulation of rCBF in M1-HAND during short-duration aTDCS

We found no change in the regional perfusion level during our 30 s blocks of aTDCS compared to baseline. Because our main interest was to map individual dose–response of TDCS, we aimed to measure the immediate effect of regional neuronal response, in contrast to secondary prolonged aftereffects. We expected that the short 30 s of TDCS would be sufficient to create an acute increase in rCBF, because early electrophysiological studies on animals have showed that short direct polarization of the cortex (0.2 s repetitive and 5–10 s periods) with electrical stimulation can induce acute changes in spontaneous neural firing rate^27–29^. Previous preclinical rat studies also confirmed acute vascular response to short duration TES using Laser-Doppler Flowmetry (LDF) with immediate frequency and intensity dependent vasodilation of the middle meningeal artery up to 140% of baseline after 10–30 s of rectangular pulse waves on the skull^32,33^ and dura^31^. Functional near-infrared spectroscopy (fNIRS) has also measured immediate increase in Oxy-Hb concentration following 200 μA aTDCS, applied 10 min over the right barrel cortex, and a decrease right after stimulation terminated^46^.

As we used a different imaging technique to probe the neural and vascular response, this might account for the lack of reproducibility of these previous blood flow findings. However, our negative findings appear to be at variance with recent ASL-MRI TDCS studies that reported a regional increase in cortical perfusion underneath the electrode. Previous studies used markedly longer stimulation blocks, lasting 8 or 20 min^40,41^. Therefore, it is likely that prolonged TDCS protocols may result in more robust and consistent effects on rCBF that will be traceable with fMRI-ASL. The effects on rCBF produced by longer TDCS protocols may not primarily be caused by the direct depolarizing effects of TDCS, reflecting the TDCS-induced shift in neural activity. Changes in rCBF after prolonged TDCS may rather be driven by secondary neuromodulatory effects of TDCS^47,48^. If so, ASL may be more reliable in capturing TDCS-induced changes in rCBF that are caused by longer lasting, secondary neuronal responses.

The absence of reliable rCBF changes in response to 30 s periods of aTDCS also raises the question whether short protocols are sufficient to shift corticomotor excitability in the targeted M1-HAND at all. Here it is important to note that TMS of M1-HAND did reveal a significant increase in cortical excitability already after a short period (i.e., 4 s) of low intensity (1 mA) aTDCS^11^. As we did not find a comparable immediate increase in regional perfusion, we hypothesize that the rCBF response probed with ASL is also less responsive to the polarization effects of TDCS than the pyramidal cortico-spinal neurons, that are trans-synaptically probed with TMS.

### Robust rCBF increase in left M1-HAND during finger tapping

While we were not able to detect stimulation-related rCBF changes, M1-HAND showed a robust motor-task induced increase in rCBF during contralateral finger tapping. This indicates that our pcASL measurements were sensitive to increases in regional neuronal activity. When using a more liberal threshold, there was also a trend towards a decrease in regional perfusion in the ipsilateral (right) primary sensory cortex. The task-induced ipsilateral perfusion decrease has not been studied with ASL previously but supports previous neurophysiological findings on interhemispheric interaction^49^, and could be utilized by future studies to investigate disruptions of the interhemispheric network during conditions like stroke. Together, the demonstration of task-related bi-directional changes in M1-HAND activity suggests that the lack of any stimulation-induced effects on rCBF in M1-HAND cannot be attributed to an inability of our ASL sequence to detect perfusion changes.

Comparing the perfusion during finger tapping before and after the stimulation blocks suggested that the four aTDCS blocks did not induce a change in rCBF that outlasted stimulation. This is consistent with previous BOLD fMRI studies, showing no significant change of task-related activation after consecutive blocks of 20 s TDCS^50^ and 5 min TDCS^51^.

### No dose-dependent changes in rCBF during short-duration TDCS

We did not find any intensity-related difference in rCBF levels during short-duration aTDCS. Our focus on the influence of stimulation intensity on TDCS-induced changes in regional neuronal activity is motivated by previous dose-dependent TMS studies, showing a sigmoidal dose–response curve in stimulation intensity and evoked motor response and motor evoked potential^52–54^. There is still a great deal of uncertainty regarding which biophysical and neurobiological mechanisms contribute to the neuromodulatory effects of TDCS and how they interact with each other. Although we expect an increase in neuromodulation with current intensity, this remains to be shown. Currently, it is difficult to predict the shape the dose–response relationship. Since the neurophysiological impact of TDCS will be influenced by multiple neurobiological factors^55^, the dose–response relationship between stimulation intensity and the induced change in regional neuronal activity is likely non-linear. A non-linear relationship between changes in neuronal activity and the current intensity of TDCS has been suggested previously^56,57^, with lower currents potentially inducing stronger effects than high currents^58^. Of note, we found a small but consistent effect of low-intensity aTDCS at 0.5 mA on rCBF in the deep region of M1-HAND (Fig. 2, Supplementary Fig. 4E). We find this activity-enhancing effect at the lowest current intensity interesting, as it may reflect stochastic resonance effects, which can emerge at very weak electric fields^55^. As the sample size is rather small, we cannot exclude a false-positive finding, and this effect should be replicated in future studies for verification.

We did not apply aTDCS at intensities above 2 mA. Therefore, it is possible that higher current intensities might be needed for causing a consistent change in rCBF with short-duration aTDCS. This hypothesis is supported by recent findings from Jonker et al. who did not find any effect on cortical excitability after 20 min of aTDCS at 2 mA^16^ together with in vivo measurements of neural spiking activity in animals^20^ and humans^19,59^. These measurements suggest that convential TDCS at 2 mA current induces electrical field gradients at the lower end of needed to induce neuronal spiking activity. Our negative results thus emphasizes the relevance of exploring the effect of TDCS at higher current intensities in future experiments.

### Difference in target engagement in sub-regional VOIs

Recent biophysical models suggest that the effect of the induced current is dependent on the orientation of the targeted neurons. Pyramidal tract neurons have lower thresholds for activation at the axon terminal and node of Ranvier^60^, and the depolarization of these segments is highly dependent on their orientation to the electric field: Neurons in the gyrus crown are oriented perpendicular to the E-field and depolarization will predominantly occur at the proximal part of the axon^60,61^, whereas neurons located in the sulcal depth will not achieve the same level of polarization. Different susceptibility to electric stimulation in superficial versus deeper structures is supported by E-field simulations showing that the induced electrical field is highest at the gyral crown^62^. We therefore expected higher engagement of aTDCS in the superficial M1. Contrary to our hypothesis, aTDCS did not induce regional-specific perfusion changes, neither in the superficial M1-HAND nor in deeper regions or the adjacent PMd. The inability to detect any region-specific effects on rCBF might be attributed to the low current intensities and the use of a classical non-focal electrode montage.

### E-field simulations of target engagement

Our E-field simulations revealed that bipolar aTDCS induced its maximum E-field more rostrally in the frontal cortex than the intended stimulation target (M1). There was however no significant aTDCS induced change in rCBF in the area of maximal current density either. At the group level, the peak E-field location was located in the left dorsolateral prefrontal cortex. This finding is consistent with previous work showing that the maximum TDCS-induced E-field is not necessarily located directly underneath the stimulation electrodes^63^.

In a recent ASL-TDCS study, Jamil et al. reported that 15 min of bipolar TDCS targeting M1 induced polarity-specific changes in rCBF under the M1-electrode, together with regional E-field dose-dependency with the strongest correlation at TDCS intensities between 1 and 2 mA^43^. In our study, we used the same montage and intensity range, but applied short period TDCS. The short periods of TDCS showed no correlation between the regional induced E-fields and rCBF. Interestingly, the individually induced E-field in the deep region of M1-HAND predicted the rCBF increase during low-intensity aTDCS at 0.5 mA. This finding suggests that for short lasting TDCS, the induced E-field scales with the neuronal activation in the deeper part of M1-HAND in a dose-dependent manner only at low current strength. Stochastic resonance may explain why variations within an apparently narrow range of weak currents positively correlate with the regional neural response^55,64^.

In summary, We consistently found that the immediate perfusion responses to short-duration aTDCS are highly variable among subjects (as presented in Fig. 4). On the one hand, this inter-individual variability challenges the common practice to apply a fixed stimulation intensity in TDCS studies, as this will most likely evoke substantially different physiological responses across individuals. On the other hand, the fact that we could not find a clear E-field-dependent change in rCBF, indicates that our setup may not be the best feasible method to define individual dose–response relationships and to guide the personalized dosing of TDCS.

### Limitations

We used pcASL-MRI to measure changes in regional perfusion as proxy read-out of regional neural activity. Since the ASL signal is noisy, we may have missed subtle changes in regional perfusion. Since our pcASL MRI approach reliably detected movement related activation of M1-HAND in each individual, we argue that our approach was sensitive enough to detect task-related fluctuations in regional neuronal activity.

The experimental design did not include a sham condition to control for off-target effects of aTDCS for instance due to somatosensory co-stimulation. We also did not test for polarity dependent effects, as we only targeted the M1-HAND with anodal stimulation. However, as we have not found any significant changes in regional cortical perfusion related to stimulation, we argue that this does not reduce the reliability of the results. The nine participants in this study showed highly variable cortical perfusion patterns during TDCS. Apart from a subtle increase in regional cortical perfusion at the lowest intensity level, a short period of bipolar aTDCS failed to induce a further increase in cortical perfusion at higher current intensities. We thus conclude that our TDCS-ASL approach cannot be used to inform personalized dose adjustment in TDCS studies.

Our study captured individual dose–response profiles and demonstrated substantial inter-individual variability. Although we found no consistent dose–response relationship between current intensity of aTDCS and regional cortical perfusion, the low number of participants precludes any firm conclusions at the group level. On the one hand, the small sample size may have introduced a bias towards detecting false-positive spurious findings (e.g., the mild increase in perfusion at the lowest stimulus intensity). On the other hand, the small sample size bears an inherent risk for missing out subtle differences in regional cortex perfusion among stimulation conditions due to reduced statistical power. Future studies aimed at target engagement and personalization of TDCS may focus more on focal electrode montages^65^, taking individual anatomy into consideration for electrode placement^62^, and investigating the tolerability and effect of higher current intensities^19,20^.

## Conclusion

We show that pcASL MRI does not reveal consistent immediate effects of short-duration aTDCS on neural activity, but that it reliably picks up activity changes during a simple unimanual task. The motor task was associated with a perfusion increase in the contralateral M1-HAND along with a concurrent decrease in perfusion in the M1-HAND ipsilateral to the moving hand. This discrepancy suggests that the immediate changes in regional neural activity during short periods of aTDCS may be too subtle to be detected with pcASL. We therefore conclude that our approach is not suited for mapping dose–response profiles using short periods in this manner. It is possible that more robust results may be achieved by performing pcASL MRI during longer administration periods of aTDCS. This would also better match to TDCS protocols that are more commonly used in clinical and research settings. Longer periods of aTDCS might produce more substantial shifts in regional neuronal activity leading to clearer changes in rCBF. One should also note, that such changes might have a primary neural or vascular origin, given that TDCS has primary effects on both the neural and vascular structures of the brain^66^.

## Materials and methods

### Subjects

Nine young healthy individuals participated in this study (mean age 31.22; SD 4.55; 5 males). All subjects were right handed, as determined by the Edinburgh Handedness Inventory^67^ with a mean laterality quotient of 97. Subjects were recruited from an open access advertisement posted on a website for subject recruitment (http://www.forsøgsperson.dk). All subjects gave their written informed consent. The experimental protocol (H-18031987) has been approved by the Regional Committee on Health Research Ethics of the Capital Region of Denmark, and the Declaration of Helsinki. All methods were performed in accordance with approved institutional guidelines and regulations.

As described in the introduction, several combined TMS-PET studies found robust increases in rCBF in small groups of healthy individuals^25,26,68^. Since dose–response profiles were consistently expressed at the subject level, we reasoned that a sample size of nine individuals would be sufficient to test our two hypotheses that regional perfusion in the stimulated M1-HAND would increase with the intensity of TDCS and that this response pattern would be consistently expressed among healthy participants.

### Study design

**Figure 5:**
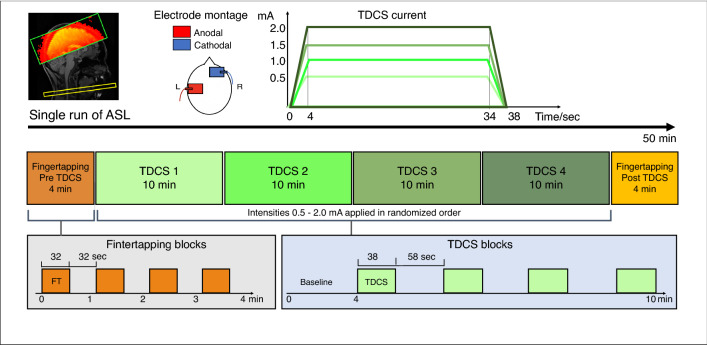
Experimental design. Top: TDCS was applied with 5 × 7 cm^2^ square electrodes with anodal on C4 and cathodal on the right supraorbital area. We applied four different current intensities of 0.5, 1.0, 1.5 and 2.0 mA. Current waveform consisted of 4 s ramp-up, 30 s stimulation and 4 s ramp-down. Middle and bottom: We performed a single run of pcASL, with a block of 4 min finger-tapping (FT) followed by four consecutive 10 min blocks of stimulation (TDCS) with randomized order of intensities, ending with a block of 4 min FT. Total duration of the scanning session is approximately 50 min.

Participants underwent a single pcASL-MRI session that lasted approximately 50 min. The experimental procedures are illustrated in Fig. 5. Bipolar aTDCS was applied with the anode targeting left primary motor cortex (M1) and the cathode on the right side of the forehead (supraorbital (SO) region). During stimulation, participants were resting in the MRI scanner with their eyes focusing on a fixation cross displayed in the middle of a screen.

The experiment included four aTDCS blocks. During a TDCS block, one of four current intensities were applied. Target current intensity was set at 0.5 mA, 1.0 mA, 1.5 mA or 2.0 mA in a given block. The order of blocks was pseudorandomized and participants were blinded. Each aTDCS block lasted 10 min and consisted of 4 min no-stimulation baseline followed by alternating epochs of aTDCS (38 s) and periods without TDCS (58 s). During an aTDCS epoch, stimulation current was linearly ramped up to the target intensity within 4 s, continuously applied at target intensity for 30 s, and then ramped down again within 4 s.

After each aTDCS block, the participants answered a series of questions through the MR-speaker system about how they had experienced the preceding aTDCS block, using a six-level Visual Analogue Scale (VAS) (see Supplementary Table 1).

We included finger-tapping (FT) blocks without stimulation at the beginning (FT-pre) and the end (FT-post) of the pcASL-MRI experiment to compare rCBF changes evoked by aTDCS with rCBF change during voluntary motor activity. During FT blocks participants tapped their right (dominant) index and thumb together paced by a blinking cross (2 Hz). Each FT-block was four min, consisting of interleaved 32 s epochs of FT and 32 s of rest.

### Transcranial DC stimulation in the MRI scanner

We applied aTDCS at 0.5, 1.0, 1.5 and 2.0 mA. The center of the anodal electrode corresponding to the C3 location of the 10/20 EEG system^69^, with 2-mm thick 7 × 5 cm rubber electrodes (NeuroConn, Illmenau, Germany). Connector plugs were arranged pointing down towards the ear for the anodal electrode, and horizontally outwards for the cathodal electrode. Electrodes were applied with a thin layer of ten-20 conductive gel (Weaver and Company, Aurora, Colorado, US) and fixated with a net cap. Current was applied by a battery-operated DC-stimulator with MR compatible stimulation cables and filter-boxes (NeuroConn Illmenau, Germany). For safety reasons, the total impedance was kept below 15 k$\Omega$, which included the two 5 k$\Omega$ resistors in the cables.

### MRI image acquisition

Images were acquired on a Phillips 3 Tesla MR Achieva scanner (Philips, Best, Netherlands) using a 32-channel head coil. A localizer scan assessed the head-position prior to a structural T1-weighted whole-brain scan using a 3d-TFE multi-shot sequence (TR/TE = 6.0/2.7 ms; flip angle = 8°; FOV = 245 FH 245 AP 208 RL mm^3^; isotropic resolution = 0.85 mm^3^) and a T2-weighted whole-brain scan using a 3D-TFE multi-shot sequence (TR/TE = 2500/265 ms; flip angle = 90°; FOV = 245 FH 245 AP 190 RL mm^3^; isotropic resolution = 0.85 mm^3^).

Pseudo-continuous (pc)ASL was acquired as a single run per block, resulting in six ASL-MRI runs per participants, corresponding to the four aTDCS and two FT blocks per participant (Fig. 5). The labeling plane was positioned where the Vertebral and Internal Carotic Artery are parallel (approximately at C2 level) and angled perpendicular to the vessel orientations. pcASL images were acquired with background suppression by two pulses, pulsed continuous labeling, label duration of 1650 ms, and post-label-duration of 1200 ms, using an echoplanar imaging (EPI) readout. Image resolution was 3 × 3 × 4 mm. A single ASL volume consisted of 17 slices with a gap of 0.5 mm, covering the pericentral cortices and adjacent frontoparietal regions of both cerebral hemispheres. Duration for each ASL dynamic was 2 × 4.0 s, with 78 dynamics per aTDCS block, and 32 dynamics per FT block.

### Data analysis

All ASL-MRI data was analyzed using FSL software, Wellcome Centre for Integrative Neuroimaging, University of Oxford. (https://www.win.ox.ac.uk).

Pre-processing of ASL-MRI data is described in Appendix A. ASL-MRI data were analyzed using FSL FEAT. For each participant, we did six separate first level analyses, one for each of the four aTDCS runs, and two for FT-runs. Voxel-wise changes in rCBF were analyzed by fitting a General Linear Model (GLM) to the time series of each ASL-MRI run/block modelling either one of the four aTDCS intensities or the movement sequences. The GLM featured three regressors, as previously described in Moisa et al.^70^ (further details describing our GLM in Appendix A). The positive z-activation map from the perfusion regressor was used for group level analyses, described below.

### Definition of volumes of interests

The left M1-HAND was our primary volume of interest (VOI). We also wanted to determine specific functional changes in sub-regions within the primary VOI, based on depth and proximity to the anodal electrode and we planned to include the region with the highest induced E-field as an additional VOI (for an overview of VOI abbreviations and definitions, please see Table 3):

**Table 3:** Overview of VOI's.

Abbreviation	Definition	Coordinate of peak voxel
**Primary VOI's**		
M1_FT_	Average activation cluster from finger-tapping pre-TDCS (FTpre)	− 38, − 24, 60 Cluster size = 325 voxels
M1-S1_anat_	Based on probabilistic atlas (FSL Juelich) areas BA4a, BA4b BA3a, BA3b and BA6 for the left hemisphere	

Abbreviation	Definition	Coordinate of center of sphere
**Secondary VOI's: 10 mm spheres**		
M1_deep_	Center placed at the deep point of highest regional activation in the primary motor cortex from from FTpre activation	− 38, − 24, 46
M1_superficial_	Center placed at the superficial point of highest regional activation in the primary motor cortex from FTpre activation	− 38, − 24, 60
PMd	Center placed in a functional activated area in the dorsal premotor cortex (area 6 in Juelich atlas) from FTpre activation	− 38, − 6, 60
DLPFC_E-field_	Center placed on the gray matter location of maximum averaged E-field. Corresponding to the left dorsolateral prefrontal cortex	− 40, 28, 42

#### Functional defined VOI of the left M1-HAND (M1_FT_)

We used the average FT-pre activation as a functional localizer for this VOI (illustrated in Figs. 2, 4A, and Supplementary Fig. 2). The VOI was derived from a whole-brain voxel-wise group analysis using a one-sided t-test with a corrected statistical threshold of p < 0.05 set for family wise error (FWE) cluster level correction and a cluster-defining threshold that was set to p < 0.001 at the voxel level (corresponding to Z = 3.1).

#### Anatomical defined VOI of the left primary sensorimotor cortex (M1-S1_anat_)

To avoid bias from the motor-task guided VOI (M1_FT_), we additionally defined an anatomical VOI around the left primary sensory-motor cortex based on the probabilistic atlas from FSL’s Juelich map, including areas BA4a, BA4b BA3a, BA3b and BA6 for the left hemisphere. The probabilistic area masks were thresholded at 50% relative to their peak values and binarized. In addition, the extend of the mask was restricted to fit area around M1-HAND by including only voxels with MNI coordinates between − 52 mm and − 25 mm in left–right direction and ≥ 36 mm in inferior-posterior direction (Fig. 3).

#### Sub-regional spherical VOI’s in precentral cortex (M1_deep_, M1_superficial_, PMd)

Secondary VOI analyses included three precentral sub-regions and the frontal cortical site that was exposed to the maximal electrical field during aTDCS. We defined three VOIs within the left pre-central gyrus (10 mm spheres) using the FT-pre as a functional localizer. The VOIs were placed at the regional activity peaks within M1_FT_, (Fig. 4A). Two of the VOIs were placed at the superficial and deep point of highest regional activation in the primary motor cortex, “M1_deep_” and “M1_superficial_” (center coordinates x, y, z: [− 38, − 24, 46] and [− 38, − 24, 60]). The third VOI was placed in a functional activated area corresponding to area 6 in the Juelich atlas in FSL, the dorsal premotor cortex “PMd” (center coordinate [− 38, − 6, 60]).

#### Computational modeling of electric field

We performed additional analysis that considered the current distribution in each subject. We used individual structural scans to simulate the induced electric field for each subject, using SimNIBS v.3.2.2^21^. The individual E-field maps were non-linearly transformed to MNI space by applying the deformation field to the data that was calculated by SPM12 during the head modeling with headreco. For further details on E-field simulations, please see Appendix A (and Fig. 4).

#### E-field guided VOI (DLPFC_E-field_)

To define the VOI for the maximal electrical field, each individual E-field simulation at 1 mA were overlaid in MNI-space, considering only positions at which the gray matter of a least 5 subjects overlapped. A 10 mm VOI was placed at the gray matter location at maximum of the averaged E-field (sphere center coordinate: − 40, 28, 42), corresponding to the left dorsolateral prefrontal cortex (DLPFC) (bottom right panel in Supplementary Fig. 3).

### Statistical inference on group level

#### Regional perfusion changes in the cortical VOIs

We ran six GLM analyses at group level, one for each aTDCS condition, corresponding to the four aTDCS intensities, and the two finger tapping sessions. The parameter estimates from the perfusion regressor from the first level analyses was fitted into a second level GLM that modelled the group average, corresponding to a one-sided t-test for each condition. In each model, the z-scores were averaged within our pre-defined volumes of interest (VOI definitions described in “Definition of volumes of interests”). For each VOI, we submitted the spatially averaged z-scores into a two-way repeated measure ANOVA for within subject factor “conditions” with six levels (four aTDCS intensities, two movement sequences). We performed post-hoc tests with pairwise comparisons, p-values were corrected for multiple comparisons using the Bonferroni method. For all ANOVA’s, we used Mauchly’s test to test for sphericity, and corrected with the Greenhouse Geisser method when sphericity was violated. ANOVA and post hoc tests were done using version 25 of the SPSS statistics software package (IBM, Armonk, New York, USA).

#### Correlation between regional E-field and regional perfusion change

We explored the relation between the highest induced E-field and perfusion response on an individual level. Here we simulated the E-field (at 0.5, 1.0, 1.5, 2.0 mA) in all four VOIs (M1_deep_, M1_superficial_, PMd, DLPFC_E-field_) in all subjects. For each intensity and each VOI, we ran linear regression analyses on the mean perfusion activation and E-field, to see if there were any correlation between the individual perfusion changes and estimated individual E-field strength (see Supplementary Fig. 4). We used Bonferroni correction between the 16 comparisons, with an alpha level of 0.05.

#### Voxel-based exploratory analyses

We performed a complementary voxel-based analysis to test for linear increase or decrease in perfusion throughout all aTDCS intensities, including all voxels. We applied a cluster-size threshold of p = 0.05 and voxel-wise threshold of z = 3.1 or p = 0.001. We additionally explored finding potential activity with a more liberal voxel-wise threshold of z = 2.3 or p = 0.01.

#### Analysis of psychometric data

For details on analysis of VAS-scale rated sensory experience, please see Appendix A.

## Supplementary Information

Supplementary Information.

## Data Availability

The datasets generated and/or analysed during the current study are not currently publicly available as they require complete anonymisation but will be available from the corresponding author on request.
